# Benchmarking ortholog identification methods using functional genomics data

**DOI:** 10.1186/gb-2006-7-4-r31

**Published:** 2006-04-13

**Authors:** Tim Hulsen, Martijn A Huynen, Jacob de Vlieg, Peter MA Groenen

**Affiliations:** 1 Centre for Molecular and Biomolecular Informatics, Nijmegen Centre for Molecular Life Sciences, Radboud University Nijmegen, Toernooiveld 1, Nijmegen, 6500 GL, The Netherlands; 2 NV Organon, Molenstraat 110, Oss, 5340 BH, The Netherlands

## Abstract

**Background:**

The transfer of functional annotations from model organism proteins to human proteins is one of the main applications of comparative genomics. Various methods are used to analyze cross-species orthologous relationships according to an operational definition of orthology. Often the definition of orthology is incorrectly interpreted as a prediction of proteins that are functionally equivalent across species, while in fact it only defines the existence of a common ancestor for a gene in different species. However, it has been demonstrated that orthologs often reveal significant functional similarity. Therefore, the quality of the orthology prediction is an important factor in the transfer of functional annotations (and other related information). To identify protein pairs with the highest possible functional similarity, it is important to qualify ortholog identification methods.

**Results:**

To measure the similarity in function of proteins from different species we used functional genomics data, such as expression data and protein interaction data. We tested several of the most popular ortholog identification methods. In general, we observed a sensitivity/selectivity trade-off: the functional similarity scores per orthologous pair of sequences become higher when the number of proteins included in the ortholog groups decreases.

**Conclusion:**

By combining the sensitivity and the selectivity into an overall score, we show that the InParanoid program is the best ortholog identification method in terms of identifying functionally equivalent proteins.

## Abstract 2

A benchmarking of the most popular orthologous identification methods using functional genomics data identifies the two best methods.

## Background

Orthology is one of the central concepts of comparative genome analysis, but is often misused as a description of functionally equivalent genes in different species. By definition, the term describes the evolutionary relationship between homologous genes whose independent evolution reflects a speciation event, whereas paralogy refers to genes that have diverged from a common ancestor through a gene duplication event [1]. Orthologous genes are more likely to have a functional similarity than paralogous genes, which have often undergone changes in substrate or ligand specificity [2,3]. The high level of functional conservation between orthologous proteins makes orthology highly relevant for protein function prediction. It is also widely used in genome analysis, where the information about a protein in one species is used for the functional annotation of the orthologous protein in another species. At the level of protein-protein interactions, for example, it allows networks of orthologous sequences to be investigated to detect conservation of processes and pathways.

So far, the genomes from more than 200 organisms have been fully sequenced. Of particular interest for medical research are the full genome sequences of human and model organisms, such as fruit fly, worm, mouse, rat, and chicken. Genome sequencing projects on other model organisms, such as the chimpanzee [4], are also close to completion. Identification of orthologous relationships between these model organisms and human allows the functional annotation of a model organism protein to be transferred to its human ortholog.

Given the large amount of data, automated determination of orthology relations is an absolute requirement for an optimal knowledge transfer between the proteins and pathways from different species. Several ortholog identification methods have been described that use sequence comparisons, for example, Clusters of Orthologous Groups (COG) [5], InParanoid [6] and OrthoMCL [7]. One of the most striking differences between the various methods and databases is the level of inclusiveness: the number of proteins from one species that is considered to be part of the same orthologous group. For the best bidirectional hit (BBH) method this number is one, except for theoretical cases where two proteins from species A have the same score to a protein from species B or when one considers fusion or fission of genes [8]. In the euKaryotic Orthologous Groups (KOG) database [9], this number can easily become larger than 100 proteins, for example, for trypsin (KOG3627) in *Homo sapiens*. The reasons for this difference in inclusiveness are twofold. Firstly, there are differences between the algorithms being employed, such as bidirectional best hits, the triangular best-bidirectional hits scheme of the COGs [5], the graph-clustering program OrthoMCL [7], the sequence similarity based InParanoid [6], or a phylogenetic tree algorithm [10]. Secondly, some databases include a wider phylogenetic array of species than others. To give one example, the KOG database [9] aims to include all sequenced eukaryotes. In such a situation, genes resulting from relatively recent gene duplications, like those in the lineage leading to the mammals, will all be part of the same orthologous group. In a database that includes only the mammals, for example, a version of InParanoid that compares mouse and human, these genes will likely be split into different orthologous groups. Comparing only recently diverged species, therefore, allows one to obtain a higher level of evolutionary, and possibly also functional, resolution.

The various published orthology identification methods have led to the recognition that it would be useful to compare these algorithms and use the consistency in the predicted orthologous relations as a measure of reliability [11]. Additionally, several procedures have been proposed to test the reliability of orthology prediction from a single method [6,12]. It has even been proposed that one could actually use functional genomics data to assess the reliability of orthology prediction algorithms to predict functional equivalent genes [13]. However, consistency in the prediction is no measure of statistical or biological significance and the comparison of several ortholog identification methods using functional genomics data is, to the best of our knowledge, a complete new approach to the problem. Here we define and follow a strategy to test the quality of several currently used ortholog identification methods to identify functionally equivalent proteins. Unfortunately, there is no 'gold standard' of protein function that can be used to benchmark ortholog identification methods, as experimentally determined functions are only known for a very small fraction of the proteins in the sequenced genomes. Hence, assessing the quality of different methods currently used is not a straightforward exercise. In our strategy, we use the assumption that functionally equivalent orthologs should behave similarly in functional genomics data [14]. This aspect of conservation of function can be measured in several ways: by similar expression profiles (tissue distribution or regulation), conservation of co-expression, identical domain annotation, conservation of protein-protein interaction or involvement in similar processes (pathways). All of these properties are used here to benchmark the quality of several commonly used ortholog identification methods. The outcome of this benchmark will be useful for determining which ortholog identification method should be used to identify orthologous relationships. Moreover, it gives an idea of which methods are good at predicting different kinds of functional conservation. Some methods appear to be good at predicting conservation of co-expression, while others more accurately predict the conservation of the molecular function. Which ortholog identification method one should use depends on the kind of functional annotation that is to be transferred from one protein to the other. Here we show some examples of the differences between the various kinds of functional conservation in relation to the type of ortholog identification. As a start for building a 'gold standard' of protein function, we also included a comparison with a reference set of 'true orthologs' consisting of five well-studied protein families.

## Results

### Direct conservation of functional parameters

**Figure 1:**
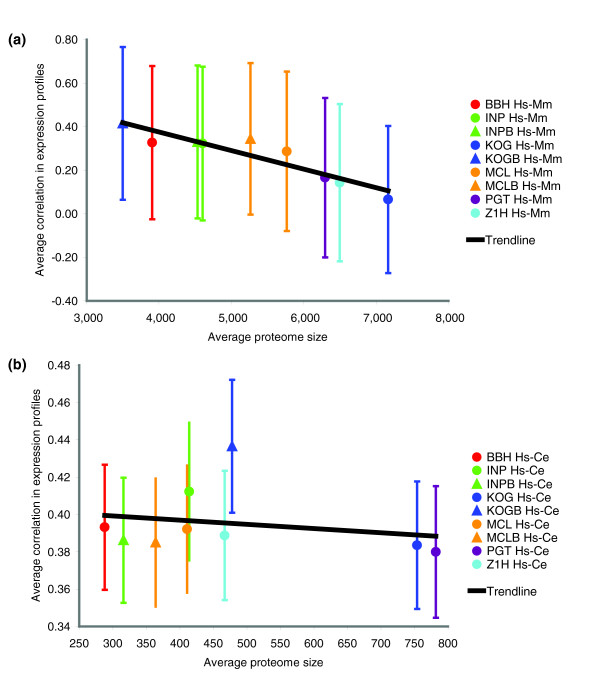
Correlation in expression profiles. Correlation in expression patterns between the **(a) **human-mouse (Hs-Mm) and **(b) **human-worm (Hs-Ce) orthologous pairs from the benchmarked methods versus the average proteome size. Vertical error bars show the standard deviation from the average correlation coefficient. The trendline shown is a linear regression trendline. The methods having a fourth letter 'B' behind the method name, shown as squares in the graph, are group orthology methods in which only the best scoring pairs are taken into account.

**Figure 2:**
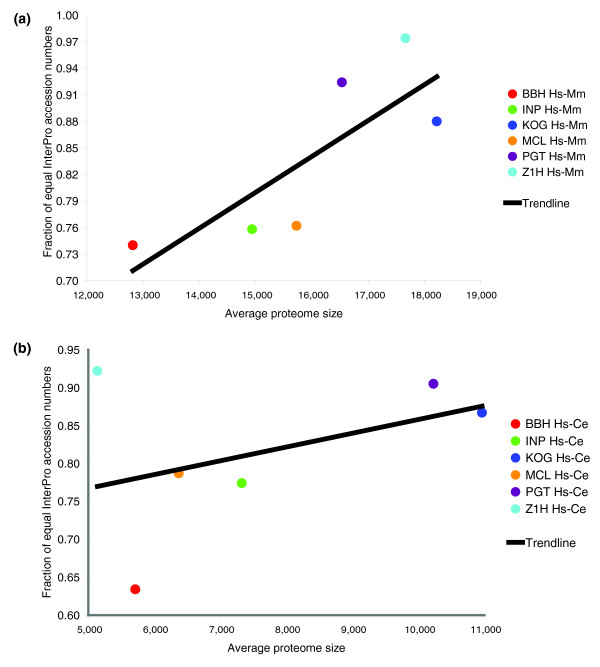
Equal InterPro accession number. Conservation of InterPro accession number between the **(a) **human-mouse (Hs-Mm) and **(b) **human-worm (Hs-Ce) orthologous pairs from the benchmarked methods versus the average proteome size.

First, we measured the conservation of functional parameters between orthologous proteins, examining direct correspondence between human and mouse/worm proteins (Figures 1 and 2). This conservation was measured by comparing the expression profiles that provide information about the functional context of a protein (Figure 1) and the InterPro accession numbers, which provide information about the molecular function of a protein (Figure 2). We determined the correlation in tissue expression patterns between the human-mouse and human-worm orthologous pairs from the six benchmarked methods (Figure 1). Note that only proteins for which gene expression data exist are included in this analysis. This is shown by the lower average proteome sizes in, especially, the human-worm analysis, for which it was difficult to map the expression data to the Protein World data. For the human-mouse analysis, this was less difficult. For the three group orthology methods, InParanoid (INP), KOG and OrthoMCL (MCL), a second calculation method was used, which only takes into account the best scoring pair within a group. An examination of only the average correlation shows that the KOG best scoring pair (KOGB) human-mouse set, containing the best scoring human-mouse pair of each KOG, seems to have the highest conservation of function. However, this set has the lowest average proteome size for human-mouse, thus combining a high selectivity with a low sensitivity. If orthology relationships between a larger number of proteins are required, the MCL and MCL best scoring pair (MCLB) sets are good alternatives. Finally, the large standard deviations are a reason to be careful with the interpretation of these results. We do not have this statistical issue when examining the conservation of InterPro accession numbers (Figure 2). The ortholog identification methods that create the most orthologous relationships have a larger fraction of equal InterPro accession numbers than the others. The many-to-many non-group methods PhyloGenetic Tree (PGT) and Z 1 Hundred (Z1H) show particularly good scores. Note that these methods use a Smith-Waterman calculation in combination with a Z-value threshold (Monte-Carlo statistics) to define the orthologous relationships (Z ≥ 20 with some additional steps for PGT, Z ≥ 100 for Z1H), whereas the methods with the lower scores, INP, KOG and MCL, use BLAST in combination with E-value statistics.

### Pairwise conservation of functional parameters

**Figure 3:**
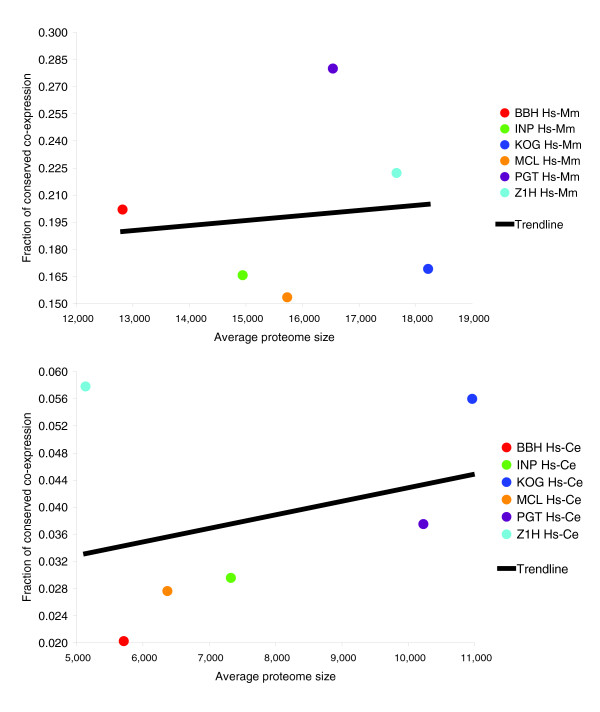
Conservation of co-expression. Conservation of co-expression from human-human gene pairs to orthologous **(a) **mouse-mouse and **(b) **worm-worm gene pairs from the benchmarked methods versus the average proteome size. Ce, *Caenorhabditis elegans*; Hs, *Homo sapiens*; Mm, *Mus musculus*.

**Figure 4:**
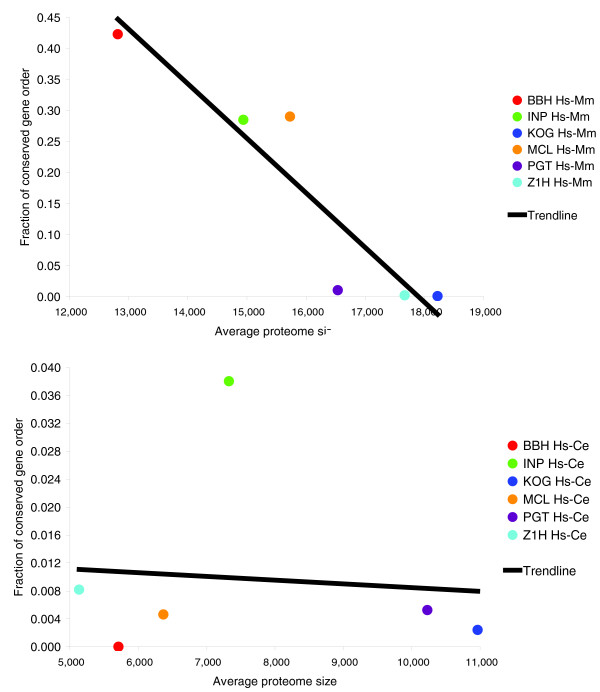
Conservation of gene order. Conservation of gene order from human-human gene pairs to orthologous **(a) **mouse-mouse and **(b) **worm-worm gene pairs from the benchmarked methods versus the average proteome size. Ce, *Caenorhabditis elegans*; Hs, *Homo sapiens*.

**Figure 5:**
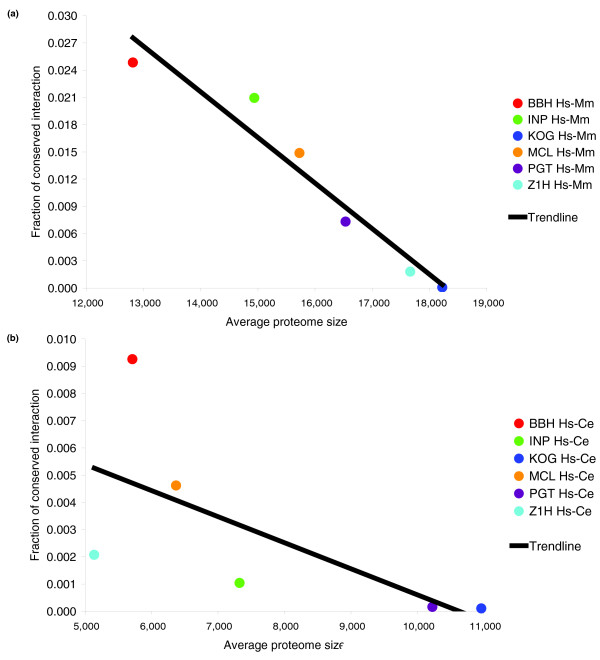
Conservation of protein-protein interaction. Conservation of protein-protein interaction from human-human protein pairs to orthologous **(a) **mouse-mouse and **(b) **worm-worm protein pairs from the benchmarked methods versus the average proteome size. Ce, *Caenorhabditis elegans*; Hs, *Homo sapiens*.

We examined three other methods for orthology prediction benchmarking. In these benchmarks, rather than comparing one-to-one functional correspondence between human and mouse/worm proteins, we compared the correspondence of the relationship between two proteins in human with the relationship between their two orthologs in mouse/worm. In this article, we refer to these methods as 'pairwise conservation of functional parameters' (Figures 3, 4 and 5). This functional conservation between two human proteins and two mouse/worm proteins is measured by comparing the co-expression levels (Figure 3), the neighboring relationships (Figure 4) and the protein-protein interactions (Figure 5) between these two species. As described in some recent papers [9,15], the evolutionary conservation of co-expression can be used for function prediction. Here it is used to test which of the ortholog sets can be used to best improve the function prediction, using the Gene Ontology (GO) database [16]. According to our first pairwise benchmark (Figure 3), the PGT approach is the best method in the human-mouse analysis, having the highest fraction of equal 4th level GO biological process and the third/fourth largest average proteome. Z1H is the second best method when using conservation of co-expression as a benchmark, having both the second highest sensitivity and the second highest selectivity. The second benchmark, the conservation of gene order, gives completely different results (Figure 4): the BBH, INP and MCL methods have the best scores. The three methods with a relatively large average proteome size (PGT, Z1H and KOG) have exceptionally low scores here: all have a fraction of conserved gene order below 0.02. For the conservation of protein-protein interaction (Figure 5), the smallest set of all, BBH, has the best score. However, the INP and MCL sets have the best score when both the fraction of conserved protein-protein interaction and the average proteome size are taken into account. Although not as dramatically low as the fractions of conserved gene order, the fractions of conserved protein-protein interaction are still quite low for the three methods with the largest average proteome size.

### Overall results

**Figure 6:**
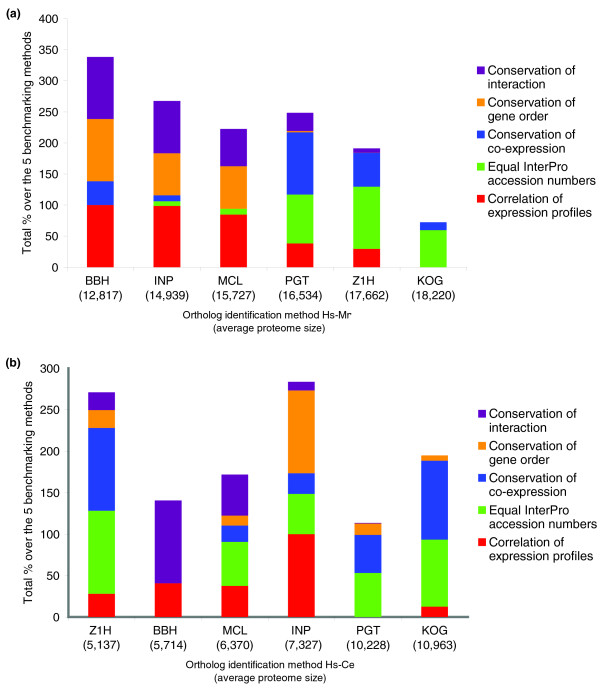
Overall scoring graph. Overall scoring graph, created by adding up all normalized benchmarking scores per ortholog identification method. X-axis, the several ortholog identification methods, sorted by average proteome size or number of protein pairs; Y-axis, the sum of all five benchmarking scores per ortholog identification method. Red, correlation of expression profiles; green, equal InterPro accession numbers; blue, conservation of co-expression; orange, conservation of gene order; purple, conservation of protein-protein interaction. **(a) **Human-mouse (Hs-Mm). **(b) **Human-worm (Hs-Ce).

From the independent results it is difficult to draw a conclusion on which method is best. We therefore determined an overall benchmark of the ortholog identification methods, which are calculated by multiplying the function similarity scores by the average proteome size (Table 1). Subsequently, the five resulting scores are combined into one overall score by multiplying them. Each benchmark has its own ranking, on a scale from 1 to 6, and an overall ranking according to the overall score. The overall scores and the overall ranking show that BBH and INP score best, closely followed by MCL. If we combine the several benchmarks into an overall score in a different way, by normalizing all benchmarking scores first (putting the lowest score at 0 and the highest score at 100) and then adding them up, the results are approximately the same (Figure 6a for human-mouse). Again, the BBH and INP methods have the best score, followed by the PGT and MCL methods. KOG has a very low overall score. PGT has both a higher score and a larger average proteome size than MCL. The human-worm analysis (Figure 6b) shows that the sensitivity/selectivity trade-off is less visible here. The INP method, which has the fourth largest selectivity, has the highest overall score. Z1H, the method with the largest selectivity, has only the second highest score. These results might be influenced, however, by the lower reliability of the human-worm expression data. When combining the results from Figure 6a and 6b, we can conclude that the InParanoid algorithm is the best ortholog identification method.

### Ortholog reference set

We included in our study a 'true ortholog' reference set, consisting of five well-studied protein families: the Hox cluster proteins and hemoglobins (human-mouse), the nuclear receptors and toll-like receptors (human-worm), and the Sm and Sm-like proteins (human-mouse plus human-worm). Table 2 shows the overlap between the orthologs defined by the six different methods and this reference set.

The human-mouse Hox cluster proteins are covered best by the PGT method: 33 out of 41 orthologous pairs are detected. The KOG method is the second best with 30 orthologous pairs, and InParanoid is third best with 28 pairs. The other three methods all find the same 26 pairs. However, the KOG and PGT methods also have a high number of false positives. When the number of orthologous pairs is divided by the average proteome size, the BBH method has the highest score, followed by PGT and INP. The nine human-mouse hemoglobin orthologous pairs are almost all detected by the Z1H method. The orthologous pairs/average proteome size ratios of the six different methods do not differ much for this family, which means that the number of detected pairs is proportional to the inclusiveness of the ortholog identification method. PGT and BBH have the best scores when looking at Sm and Sm-like proteins.

As for the human-worm nuclear receptors, the KOG method has the highest number of orthologous pairs. However, KOG has an extremely high number of false positives. When the numbers of orthologous pairs are divided by the average proteome size, the MCL method has the best performance. The Toll-like receptor family, which has only one member in *Caenorhabditis elegans *shows good results for KOG as well, together with the PGT method. For the Sm and Sm-like protein family, the MCL and INP methods have the highest orthologous pairs/average proteome size ratios.

## Discussion

We have tested the quality of a number of ortholog identification methods for protein function prediction by comparing functional genomics data from each of the proteins in a pair identified as orthologs. Orthologs should, in general, have a higher level of function conservation than paralogs. The results show that, in general, the less inclusive the method, the better it performs in terms of function similarity; in other words, there is a certain trade-off between sensitivity and selectivity. We correct for this by taking the function similarity score and multiplying it by the geometric average of the number of unique human proteins and the number of unique mouse/worm proteins within the ortholog set that is being studied (the 'average proteome size'). After multiplying these scores to obtain an overall score (giving each benchmark the same weight), we generate an overall ranking that gives equal weight to both the five different benchmarks and the sensitivity and selectivity. From the results, we conclude that the InParanoid method is the best ortholog identification method. However, some caution should be taken with the overall ranking system. First, the average proteome size now has the same weight as the function similarity score, while one of them might be considered more important than the other. We examined the effect of different weights for these two parameters (1:2 and 2:1 proportions) but did not find any large differences in the results. Second, some benchmarks may produce better results than others, which might be a reason to give different weights to the several benchmarks when combining them into an overall score. For example, the benchmark that uses GO annotations could be less reliable because some of these annotations are actually based on sequence similarity themselves. Third, recent research [17] suggests that the expression levels of physically interacting proteins coevolve. This indicates a strong connection between the third and the fifth benchmark in this study, which could be a reason to leave out one of them. However, coexpression can be the result of processes other than physical interaction only. The differences in the results we got from the two benchmarks also contributed to our decision not to exclude either one of them. Finally, it should be noted that the data we used in our human-mouse analysis was, in general, of higher quality than the data we used in our human-worm analysis. This applies especially to the gene expression data: for the human-mouse set we could use the SNOMED tissue classification, whereas for the human-worm set we found it quite hard to map the tissue samples to each other. The small numbers that were generated in the human-worm analysis also makes this analysis statistically less reliable than the human-mouse analysis.

The conclusion that can be drawn from this study is that the method that should be used to identify orthologs is in fact dependent on the research question one wants to answer using the orthologous relationships. For example, if the goal is to have one or more orthologs for a large number of proteins, one of the methods that allow many-to-many relationships (like InParanoid) should be applied. If selectivity (having as few as possible false positives) is more important than sensitivity (having as many as possible true positives) and having only one ortholog per protein is sufficient, the best bidirectional hit approach should give the best results. Although methods that include phylogenetic inferences to determine phylogenies should, in principle, be the best at establishing orthologous relationships, in practice they suffer from a number of drawbacks that methods solely based on pairwise identities do not have. It is commonplace, for example, to require positions in a sequence alignment to be present in all or most of the sequences in order to use them for deriving a phylogeny with ClustalW. Such requirements drastically reduce the amount of information that can be used to determine orthology relationships. In the absence of easily implementable solutions to this, computational shortcuts like InParanoid give, in our analysis, better results.

Finally, results could differ when different statistical significance scores (unpublished data), scoring matrices, gap penalties, and so on are used for the various alignment algorithms. We tried to minimize the effect of these parameters as much as possible by using the defaults of the several programs, but some programs might still be more suitable for identifying close orthologous relationships than others, while these others might be more appropriate for the identification of distant relationships. The differences observed between our human-mouse (closely related species) and human-worm (distantly related species) analyses support this statement. As for the human-worm analysis, the conservation of functional characteristics and gene order is significantly lower than in human-mouse. The latter is not surprising because millions of years of chromosomal rearrangements during evolution have changed the chromosomal organization significantly. As for the functional aspects, we can conclude that they have been poorly conserved whereas the protein domain organization has been well conserved.

## Conclusion

Because of the high degree of functional similarity between orthologous proteins, the quality of orthology prediction is an important factor in the transfer of functional annotation. To measure the functional similarity of proteins from different species we use functional genomics data, such as protein interaction data and expression data. In general, we observe a sensitivity/selectivity trade-off: the functional similarity scores per orthologous pair become higher when the number of proteins included in the ortholog groups decreases. This trend is more visible in the human-mouse comparison than it is in the human-worm comparison. Presumably, it gets less visible when the phylogenetic distance gets larger. By combining the sensitivity and the selectivity into an overall score, we show that the InParanoid program is the best ortholog identification method in terms of identifying functionally equivalent proteins. The method that should be used to answer a specific research question is, however, also dependent on, for example, the evolutionary distance between the studied species and the desirability of many-to-many orthologous relationships.

## Materials and methods

### 'Protein World' data set

For an unbiased comparison of all of the covered methods, the same data set was used at all times. This 'Protein World' (unpublished data) data set [18] was created by comparing all of the currently known and predicted proteins (SpTrEMBL [19], RefSeq [20], Ensembl [21]) through the Smith-Waterman algorithm [22], using Z-values to obtain a database-size independent estimate of significance [23]. The Smith-Waterman algorithm has been shown to be more sensitive [24] than its faster (non-dynamic programming) approximations, the BLAST [25] and FASTA [26] algorithms. The data set is freely available through the Center for Molecular and Biomolecular Informatics website [27]. As good expression data and other functional data were available for human, mouse and worm, we used the orthologous relationships between these three species for our study.

### Ortholog identification methods

The six ortholog identification methods covered in this study are listed below. Included are the best bidirectional hit method and five many-to-many methods. The many-to-many methods are divided into group orthology methods and non-group orthology methods. The group orthology methods, KOG [9], INP [6] and MCL [7], define several, distinct groups of orthologous genes and proteins. The two many-to-many non-group methods, PGT [10] and Z1H, do not define orthologous groups, but can still determine many-to-many orthologous relationships. Table 3 shows the numbers of orthologous groups, unique proteins and protein pairs within the several ortholog sets. The average proteome size is the geometric average of the total number of unique human proteins and the total number of unique mouse/worm proteins within the determined orthologous relationships.

#### Best bidirectional hit

The 'best bidirectional hit' (BBH) method is the most frequently applied method to determine orthologous pairs. It assumes that a cross-species protein pair in which each protein gives back the other protein as being the best hit in the whole other proteome is an orthologous pair. In this research, the best bidirectional hits were determined based on Z-values of the Protein World human-mouse and human-worm set, without a sequence similarity cutoff. In total, 12,817 human-mouse and 5,714 human-worm orthologous pairs were identified. Although the BBH method theoretically can give some many-to-many orthologs, it practically gives only one-to-one orthologous pairs.

#### InParanoid

In the INP method [6], all possible pairwise similarity scores between datasets A-A, B-B, A-B and B-A that score higher than a cutoff (bitscore ≥50, overlap ≥50%) are detected. Then the best bidirectional hits are determined and marked as potential orthologs. The in-species pairs that score higher than these orthologous pairs are marked as additional orthologs. These 'in-paralogs' get confidence values that indicate how similar they are to the main ortholog: 100% is assigned to the main ortholog and 0% is assigned to a sequence with the minimum similarity score required to be marked as in-paralog of a given group. Finally, overlapping groups of orthologs are resolved and bootstrap-based confidence values are added for all groups of orthologs. Additionally, an outgroup proteome can be used to test the significance of the in-paralog scores. InParanoid version 1.35 was downloaded [28] and the program was run using the standard parameters, except for the use of the BLOSUM80 matrix instead of the standard BLOSUM62 matrix. The BLOSUM80 matrix is more appropriate when studying protein pairs with relatively small evolutionary distances. The optional third outgroup proteome was left out. We used Paracel BLAST 1.4.9. Through the INP algorithm, 19,482 orthologous pairs were identified between human and mouse, comprising 12,610 orthologous groups; 17,011 orthologous pairs were identified between human and worm, comprising 4,135 orthologous groups.

#### euKaryotic Orthologous Groups

The KOG database [9] is the eukaryote specific version of the COG database [5]. The latter database is considered by many to be the standard orthology database of this moment. Both the COG and the KOG procedure start with an all-against-all comparison using BLAST, followed by the detection of triangles of mutually consistent, genome-specific best hits (BeTs). Subsequently triangles with a common side are merged to form crude, preliminary KOGs, after which a case-by-case analysis of each candidate KOG is carried out, among others to split fused proteins. The difference between COG and KOG lies within the last step, the manual curation. The KOG procedure pays extra attention to multi-domain proteins, which are quite common in eukaryotes. The KOG database currently consists of seven eukaryotic proteomes. A BLAST all-against-all was used to determine the corresponding KOG for each human, mouse and worm protein within the SpTrEMBL set. Orthologous relationships were determined between all human, mouse and worm proteins within a KOG. Because of the large groups that can be formed by KOGs, no less than 810,697 human-mouse orthologous protein pairs were determined, divided over 7,874 orthologous groups; 155,387 orthologous pairs were identified between human and worm, comprising 4,155 orthologous groups.

#### OrthoMCL

The MCL algorithm [7] starts with an all-against-all BLASTP, after which the reciprocal best similarity pairs between species are marked as putative orthologs and the reciprocal better similarity pairs as recent paralogs. A similarity matrix is calculated, followed by a Markov clustering [29], which determines the orthologous groups. A list of all human and mouse Ensembl protein identifiers linked to an OrthoMCL group ID was obtained from the authors. These Ensembl protein IDs were mapped to the SpTrEMBL proteome using EnsMart [30] version 19.3 [31]. Orthologous relationships were determined between all human and mouse proteins within all 7,002 groups, which gives a total of 12,625 orthologous protein pairs. The loss of defined orthologs was corrected for by calculating how many ensembl IDs mapped to an SpTrEMBL ID (57.3397%). The average proteome size of 9,018 (for human-mouse) was divided by 0.573397, giving a corrected number of proteins of 15,727. The human-worm IDs were obtained through the new OrthoMCL-DB [32]; 9,749 human-worm orthologous protein pairs were identified, comprising 4,705 orthologous groups. Because of the different mapping method, we did not need to correct the human-worm average proteome size.

#### Z 1 Hundred

Within the Z1H method, all cross-species protein pairs that have a Z-score of 100 or higher are considered to be orthologs. The Z-value estimates the statistical significance of a Smith-Waterman dynamic alignment score (SW-score) through the use of a Monte-Carlo process [23]. In this approach, selected pairs of sequences are shuffled randomly 200 times and realigned. The significance of the SW-score of a selected pair is then determined by comparing the SW-score of the selected pair with the scores for the shuffled pairs. By comparing the score with that of the shuffled sequences the method implicitly takes into account effects of sequence composition and sequence length. The Z1H set contains pairs of sequences whose SW-score is a hundred standard deviations higher than the average SW-score for the shuffled sequences. Using the Z1H method, 290,176 human-mouse and 21,509 human-worm orthologous protein pairs were identified. The algorithm does not identify distinct groups of proteins, and is, therefore, a non-group method.

#### PhyloGenetic Tree

The PGT method uses the output generated by multiple alignments and subsequent tree calculation [10] to define orthologous relationships. Although calculations like these are rather time consuming, they should give a better insight into the evolution of the studied proteins and in principle come closest to the original evolutionary definition of orthology. Orthologies were determined by grouping all proteins over the 9 eukaryotic species covered in Protein World that have a Z-value above 20 compared to one of the human proteins, and have a region of homology larger than 50% of the query length. The resulting 23,829 groups were aligned using ClustalW version 1.82 [33], and phylogenies were created using neighbor-joining [34]. For the calculation of the phylogenetic trees we only used the positions that were present in all aligned sequences, and levels of protein sequence identity were translated to evolutionary distances using the Kimura correction as implemented in ClustalW. The other parameters were set to default. After the calculations, an ortholog identification algorithm selects partitions in the tree that only include orthologs and in-paralogs to define the orthologous relationships per species pair [10]. For human and mouse, 85,848 relationships were identified. For human and worm, 49,979 relationships were identified. Because a phylogenetic tree is calculated for the homologs of every sequence, and the trees are not merged, this method is like the Z1H method, not a pure group method.

### Benchmarks

Below are a description and the workflow of the used benchmarks. The first two benchmarks measure 'direct conservation of functional parameters', that is, they examine only one protein in human and one protein in mouse/worm. The last three methods compare the relationship between two proteins in human with the relationship of their two orthologs in mouse/worm ('pairwise conservation of functional parameters').

The results of the group orthology methods were analyzed in two ways: we determined the average score for all pairwise orthology relationships within an orthologous group; and we only considered the best scoring pair within an orthologous group. The latter option obviously leads to a much higher score for the many-to-many orthology relationships. However, by including only one pair of orthologous sequences per orthologous group, that high score is balanced by a reduction in the total number of orthologous relationships (one per orthologous group). Both the number of orthologous relationships and the quality of these relationships are taken into account in the final assessment of the ortholog identification algorithms.

#### Direct conservation of functional parameters

To test the conservation of function, the Pearson correlation between the expression profiles of the proteins in an orthologous pair was calculated. The expression dataset used here [35] was a subset of pathologically normal human and mouse tissue samples from the Gene Logic BioExpress Database product [36]. Because of the small overlap of tissue categories (115 in human, 25 in mouse), the SNOMED [37] tissue categories were used to calculate the correlation coefficient (15 in human, 12 in mouse, 12 overlapping categories). The human dataset consists of 3,269 tissue samples and 44,792 cDNA fragments, the mouse dataset of 859 tissue samples and 36,701 cDNA fragments. A perfect correlation has a score of 1, a perfect anti-correlation has a score of -1. We used expression data from Stuart and colleagues [38] for the human-worm analysis, comparing tissues from both species that had similar expression profiles. For computing time-saving reasons, we used a sample of the dataset to calculate which tissues were similar: the first 10 human tissues were compared with all of the 978 worm tissues, using the first 10 metagenes defined by Stuart *et al*. The 'best hit' of the worm tissue samples for each human tissue sample was seen as corresponding tissue. These ten corresponding tissues were then used to calculate the Pearson correlation coefficients between the human and worm proteins, from which only the positive correlations were used. Proteome sizes were corrected for this by multiplying them by two, before calculating the average proteome size. For visualization reasons we displayed error bars of only one-eighth of the SD. Because of the differences between the human-mouse and human-worm expression data analyses, we emphasize that the two figures (Figures 1a and 1b) should not be compared to each other. The figures can, however, be used to compare the several ortholog identification methods within these species pairs.

The conservation of molecular function can also be benchmarked by examining whether the orthologs are in the same InterPro [39] family. Each InterPro accession number represents a protein family or domain, containing a cross-species set of homologous proteins with its own functional annotation. Proteins within an InterPro protein family have similar domain compositions. Again, the higher the percentage with equal InterPro accession numbers, the better the conservation of function. As InterPro annotation is based on similarity to predefined domains, it is not independent of sequence and cannot be used as a completely independent benchmark. It does, however, allow one to judge to what extent proteins that are regarded as orthologous actually do have the same domain composition. This is important because most automatic methods for orthology prediction, like OrthoMCL, do not require proteins to be full length homologs.

#### Pairwise conservation of functional parameters

To measure the conservation of co-expression, first the correlation between the expression profiles of each human-human gene pair was calculated. The expression dataset used was a subset of pathologically normal human and mouse tissue samples from the Gene Logic BioExpress Database product, as mentioned above. This time we used all of the 115 categories to calculate the Pearson correlation coefficient for the human-human pairs, and we calculated the Pearson correlation coefficients for the mouse-mouse gene pairs using the 25 tissue categories in mouse. Co-expression is considered conserved when the studied human gene pair having a Pearson correlation coefficient above a certain threshold has an orthologous gene pair in mouse that has a Pearson correlation coefficient above the same threshold. This threshold was varied between 0.0 and 1.0 with an interval of 0.1. Co-expression can be used to predict protein function, specifically when it is conserved in evolution [10,15]. To test which of the ortholog sets can best be used to improve co-expression based function prediction, we also determined which protein pairs were active in the same process, using the GO database [16]. Two proteins were said to be active in the same process if they shared a 4th level element of the GO biological process tree, in which the root is the 0th level element and every subsequent branch is one level higher. Finally, the fraction of the total protein set sharing this 4th level element was calculated for the several thresholds, as a measure for the sensitivity and selectivity of the ortholog identification method for function prediction by conservation of co-expression. In this analysis, GO labels such as 'undefined' were discarded. The human-worm analysis was performed in a similar way, but with the use of expression data from Stuart and colleagues [38]. For calculating reliable correlation coefficients, we only used genes here that had expression data for at least 900 out of the 1,202 human tissue samples. In worm, we used all genes having expression data for at least 500 out of the 979 tissue samples.

The conservation of gene order is the second measure of pairwise conservation. Here we examined if two genes were adjacent to each other on the genome using EnsMart [30] version 19.3 [31] for the human-mouse analysis and EnsMart version 34 for the human-worm analysis. For each of the pairs where this was the case, we examined if the orthologs in mouse/worm were also adjacent on the genome. If so, the gene order was considered to be conserved for this gene pair. Because no varying threshold is needed (two genes are adjacent or not), this is more straight-forward than measuring the conservation of co-expression. The fraction of neighboring human genes of which the orthologs in mouse/worm are also neighbors is used as a measure for the accuracy of orthology prediction.

A third measure of pairwise conservation is the conservation of protein-protein interaction. The Database of Interacting Proteins (DIP) database [40] was used to determine the protein-protein interactions in human and mouse/worm. A protein-protein interaction is considered conserved when two interacting proteins in human have orthologs in mouse/worm that are interacting too. Again, the fraction of interacting human proteins of which the orthologs in mouse/worm are interacting too is considered to be a measure for the conservation of function.

### Ortholog reference set

We defined a list of 'true ortholog pairs', for both human-mouse and human-worm, as a reference set. We chose the Hox cluster proteins and hemoglobins as a human-mouse reference set because of its well-studied evolution in vertebrates. We determined the homeobox orthologs using Figure 1 from [41]. This resulted in 41 orthologous protein pairs, consisting of 31 human proteins and 35 mouse proteins. The hemoglobin orthologs were identified with the use of Lecomte *et al*. [42], resulting in nine pairs of four human and nine mouse proteins. For human-worm, we used the analysis on nuclear receptors performed by Gissendanner *et al*. [43], resulting in 29 orthologous pairs of 22 human proteins and 18 worm proteins. A second human-worm orthology analysis was performed on the family of toll-like receptors [44], which has only one member in worm but 10 members in human. The fifth and final protein family, the Sm and Sm-like proteins [45], was analyzed for both human-mouse and human-worm orthologs. For this family we found 13 human proteins and 17 mouse proteins in 17 orthologous pairs, together with 6 human proteins and 6 worm proteins in 6 pairs.

For each of these parts of our reference set and for each of the six ortholog identification methods, we determined how many of these orthologous pairs were covered, together with the number of false positives (pairs having only the human protein or the mouse/worm protein from a reference pair). Finally, to have a fair comparison between the several ortholog identification methods, we calculated the number of orthologous pairs divided by the average proteome size.

## Additional data files

The following additional data are available with the online version of this paper. Additional data file 1 contains all end data used to create the figures. Additional data file 2 contains all of the protein pairs that are considered to be 'true orthologs' within our ortholog reference set, consisting of several protein families. The first column contains the name of the protein family, the second the human gene names and the third the mouse/worm gene names. The fourth column contains the corresponding human 'Protein World' entries, whereas the fifth column contains the mouse/worm entries. The last columns contain the orthologous protein pairs.

## Supplementary Material

Additional File 1 All end data used to create the figures. Click here for file

Additional File 2 All of the protein pairs that are considered to be 'true orthologs' within our ortholog reference set, consisting of several protein families. The first column contains the name of the protein family, the second the human gene names and the third the mouse/worm gene names. The fourth column contains the corresponding human 'Protein World' entries, whereas the fifth column contains the mouse/worm entries. The last columns contain the orthologous protein pairs. Click here for file

## Figures and Tables

**Table 1:** Benchmarking scores of ortholog identification methods

Method	Direct conservation of function		Pairwise conservation of function			Overall score
		
	Co-expression	Equal InterPro accession number	Conservation of co-expression	Conservation of gene order	Conservation of protein-protein interaction	
**Hs-Mm**						
BBH	1.28E+03 (3)	9.49E+03 (6)	2.59E+03 (4)	5.42E+03 (1)	3.18E+02 (1)	5.42E+16 (2)
INP	1.49E+03 (2)	1.13E+04 (5)	2.48E+03 (5)	4.26E+03 (3)	3.13E+02 (2)	5.57E+16 (1)
KOG	4.73E+02 (6)	1.60E+04 (2)	3.08E+03 (3)	1.42E+01 (6)	1.09E+00 (6)	3.61E+11 (6)
MCL	1.66E+03 (1)	1.20E+04 (4)	2.41E+03 (6)	4.56E+03 (2)	2.34E+02 (3)	5.10E+16 (3)
PGT	1.05E+03 (4)	1.53E+04 (3)	4.63E+03 (1)	1.73E+02 (4)	1.21E+02 (4)	1.56E+15 (4)
Z1H	9.29E+02 (5)	1.72E+04 (1)	3.93E+03 (2)	3.75E+01 (5)	3.17E+01 (5)	7.46E+13 (5)
						
**Hs-Ce**						
BBH	2.25E+03 (5)	3.62E+03 (6)	1.16E+02 (6)	0.00E+00 (6)	5.29E+01 (1)	5.00E+10 (6)
INP	3.02E+03 (3)	5.67E+03 (3)	2.17E+02 (4)	2.79E+02 (1)	7.62E+00 (4)	7.90E+12 (1)
KOG	4.20E+03 (1)	9.51E+03 (1)	6.14E+02 (1)	2.64E+01 (5)	1.17E+00 (6)	7.58E+11 (5)
MCL	2.50E+03 (4)	5.01E+03 (4)	1.76E+02 (5)	2.95E+01 (4)	2.94E+01 (2)	1.91E+12 (2)
PGT	3.89E+03 (2)	9.26E+03 (2)	3.84E+02 (2)	5.36E+01 (2)	1.65E+00 (5)	1.22E+12 (4)
Z1H	2.00E+03 (6)	4.74E+03 (5)	2.97E+02 (3)	4.20E+01 (3)	1.07E+01 (3)	1.27E+12 (3)

Ce, *Caenorhabditis elegans*; Hs, *Homo sapiens*; Mm, *Mus musculus*.

**Table 2:** Overlap with ortholog reference set

	Method	Orthologous pairs	Orthologous pairs divided by average proteome size	False positives
Hox cluster proteins (Hs, 31 unique proteins; Mm, 35 unique proteins; Hs-Mm, 41 protein pairs)	BBH	26	2.03E-03	3
	INP	28	1.87E-03	3
	KOG	30	1.65E-03	456
	MCL	26	1.65E-03	25
	PGT	33	2.00E-03	350
	Z1H	26	1.47E-03	19
				
Nuclear receptors (Hs, 22 unique proteins; Ce, 18 unique proteins; Hs-Ce, 29 protein pairs)	BBH	8	1.40E-03	2
	INP	13	1.77E-03	179
	KOG	20	1.82E-03	2,062
	MCL	13	2.04E-03	4
	PGT	11	1.08E-03	180
	Z1H	8	1.56E-03	8
				
Hemoglobins (Hs, 4 unique proteins; Mm, 9 unique proteins; Hs-Mm, 9 protein pairs)	BBH	2	1.56E-04	2
	INP	6	4.02E-04	8
	KOG	4	2.20E-04	52
	MCL	4	2.54E-04	3
	PGT	4	2.42E-04	23
	Z1H	8	4.53E-04	37
				
Toll-like receptors (Hs, 10 unique proteins; Ce, 1 unique protein; Hs-Ce, 10 protein pairs)	BBH	0	0	0
	INP	0	0	0
	KOG	10	9.12E-04	1
	MCL	0	0	0
	PGT	5	4.89E-04	86
	Z1H	0	0	0
				
Sm proteins (Hs, 13 unique proteins; Mm, 17 unique proteins; Hs-Mm, 17 protein pairs)	BBH	5	3.90E-04	8
	INP	5	3.35E-04	8
	KOG	6	3.29E-04	15
	MCL	4	2.54E-04	10
	PGT	7	4.23E-04	18
	Z1H	5	2.83E-04	4
				
Sm proteins (Hs, 6 unique proteins; Ce, 6 unique proteins; Hs-Ce, 6 protein pairs)	BBH	6	1.05E-03	0
	INP	6	8.19E-04	0
	KOG	4	3.65E-04	1
	MCL	6	9.42E-04	2
	PGT	3	2.93E-04	9
	Z1H	0	0	0

Ce, *Caenorhabditis elegans*; Hs, *Homo sapiens*; Mm, *Mus musculus*.

**Table 3:** General statistics of ortholog identification methods

Ortholog identification method	Orthologous groups	Protein pairs	Human proteins	Mouse/worm proteins	Average proteome size
**Hs-Mm**					
BBH	-	12,817	12,817	12,817	12,817
INP	12,610	19,482	15,344	14,545	14,939
KOG	7,874	810,697	20,478	15,640	18,220
MCL	7,002	12,625	16,676*	14,833*	15,727*
PGT	-	85,848	17,302	15,729	16,534
Z1H	-	290,176	19,055	16,149	17,662
					
**Hs-Ce**					
BBH	-	5,714	5,714	5,714	5,714
INP	4,135	17,011	9,282	5,784	7,327
KOG	4,155	155,387	12,249	9,812	10,963
MCL	4,705	9,749	7,028	5,774	6,370
PGT	-	49,979	12,499	8,370	10,228
Z1H	-	21,509	6,338	4,163	5,137

*Corrected for Ensembl-SpTrEMBL mapping. Ce, *Caenorhabditis elegans*; Hs, *Homo sapiens*; Mm, *Mus musculus*.
